# Sulfated Glycans Inhibit the Interaction of MERS-CoV Receptor Binding Domain with Heparin

**DOI:** 10.3390/v16020237

**Published:** 2024-02-02

**Authors:** Jiyuan Yang, Yuefan Song, Weihua Jin, Ke Xia, Grace C. Burnett, Wanjin Qiao, John T. Bates, Vitor H. Pomin, Chunyu Wang, Mingqiang Qiao, Robert J. Linhardt, Jonathan S. Dordick, Fuming Zhang

**Affiliations:** 1 The Key Laboratory of Molecular Microbiology and Technology, Ministry of Education, College of Life Sciences, Nankai University, Tianjin 300071, China; yangj25@rpi.edu (J.Y.); wanjin.qiao@mssm.edu (W.Q.); qiaomq@nankai.edu.cn (M.Q.); 2 Department of Chemistry and Chemical Biology, Center for Biotechnology and Interdisciplinary Studies, Rensselaer Polytechnic Institute, Troy, NY 12180, USA; songy11@rpi.edu (Y.S.); xiak@rpi.edu (K.X.); wangc5@rpi.edu (C.W.); linhar@rpi.edu (R.J.L.); 3 College of Biotechnology and Bioengineering, Zhejiang University of Technology, Hangzhou 310014, China; jinweihua@zjut.edu.cn; 4 Department of Cell & Molecular Biology, The University of Mississippi Medical Center, Jackson, MS 39216, USA; gburnett@umc.edu (G.C.B.); jtbates@umc.edu (J.T.B.); 5 Department of BioMolecular Sciences, Research Institute of Pharmaceutical Sciences, The University of Mississippi, Oxford, MS 38677, USA; vpomin@olemiss.edu; 6 Departments of Chemical and Biological Engineering, Rensselaer Polytechnic Institute, Troy, NY 12180, USA

**Keywords:** HSPGs, heparin, MERS-CoV, sulfated glycans, surface plasmon resonance

## Abstract

Middle East respiratory syndrome coronavirus (MERS-CoV) is a zoonotic virus with high contagion and mortality rates. Heparan sulfate proteoglycans (HSPGs) are ubiquitously expressed on the surface of mammalian cells. Owing to its high negatively charged property, heparan sulfate (HS) on the surface of host cells is used by many viruses as cofactor to facilitate viral attachment and initiate cellular entry. Therefore, inhibition of the interaction between viruses and HS could be a promising target to inhibit viral infection. In the current study, the interaction between the receptor-binding domain (RBD) of MERS-CoV and heparin was exploited to assess the inhibitory activity of various sulfated glycans such as glycosaminoglycans, marine-sourced glycans (sulfated fucans, fucosylated chondroitin sulfates, fucoidans, and rhamnan sulfate), pentosan polysulfate, and mucopolysaccharide using Surface Plasmon Resonance. We believe this study provides valuable insights for the development of sulfated glycan-based inhibitors as potential antiviral agents.

## 1. Introduction

Middle East respiratory syndrome coronavirus (MERS-CoV) was firstly discovered in Saudi Arabia in June 2012. It is a zoonotic virus which is transmitted between animals (bats and camels) and humans [1,2]. It has emerged as a significant global public health concern; as of October 2023, there were 2608 confirmed cases of MERS reported worldwide, including 938 associated deaths, resulting in a case fatality ratio of 36% [3]. Therefore, effective intervention strategies are urgently required to curb the transmission of MERS-CoV.

Similar to severe acute respiratory syndrome-related coronavirus (SARS-CoV), spike (S) protein is one of the proteins encoded by the MERS-CoV coronavirus [4]. It is an envelope-anchored trimeric protein that plays a key role in binding to receptors and facilitates the subsequent entry of the virus into host cells. The MERS-CoV S protein contains 1353 amino acids and is structured into two functional subunits. The S1 subunit comprises both an N-terminal domain and a C domain, and it possesses the potential to function as a receptor-binding domain (RBD) responsible for receptor binding. The S2 subunit is dedicated to membrane fusion and encompasses the fusion peptide (FP), extended heptad repeat 1 domain (HR1), and concise heptad repeat 2 domain (HR2) [5,6]. MERS-CoV infection engages in two pivotal biochemical processes. First, the RBD within the S protein binds to the cellular receptor dipeptidyl peptidase-4 (DPP4) located on the host cell surface [7,8,9]. Second, the S2 subunit undergoes a conformational change and inserts its FP into the plasma membrane, or it inserts into the endosomal membrane if the virion is within the endosome [5,10,11]. Third, the HR2 region associates with the HR1 to form a six-helix bundle (6-HB) fusion core [5,10]. This interaction brings the viral and cell membranes into proximity, facilitating membrane fusion. Various elements during this progression such as the RBD, DPP4, HR1, HR2, and associated proteases like human airway trypsin-like protease and transmembrane protease serine 2 emerge as potential targets for devising inhibitors targeting MERS-CoV fusion and entry [12]. Specifically, the MERS RBD from A368 to P586 within the S protein is used as a dedicated binding site for the DPP4 molecule and has stand out as a focal target for the development of MERS-CoV therapeutics [13,14].

Glycosaminoglycans (GAGs), constituting a family of linear and sulfated negatively charged polysaccharides, predominantly reside on the external surfaces of cells. Heparan sulfate (HS) is a highly prevalent type of GAG in mammals. The HS chains are covalently attached as side chains to core proteins, forming heparan sulfate proteoglycans (HSPGs) which are distributed on the surfaces of nearly all cell types and within the extracellular matrix. HSPGs serve as crucial participants in numerous cellular regulatory activities, including receptor activation and signaling, cytoskeleton assembly, extracellular matrix remodeling, endocytosis, and cell–cell crosstalk [15]. Within their vast functional repertoire, HSPGs play a key role in facilitating the invasion of host cells by a diverse range of viruses, including multiple coronaviruses (SARS-CoV, SARS-CoV-2, and MERS-CoV), flaviviruses, retroviruses (HIV), and herpesviruses [16]. They assist viruses in various stages, including attachment to the host cell, internalization, intracellular trafficking, egress, and dissemination. Since the outbreaks of the SARS-CoV and SARS-CoV-2, it has been extensively corroborated that coronaviruses utilize the S protein to bind to specific receptors on the cellular surface. Research further indicates that the S proteins of SARS-CoV and MERS-CoV exhibit comparable binding properties to those of HS, similar to the SARS-CoV-2 [17]. This observation suggests a conserved feature among these three types of coronaviruses, emphasizing HS binding as a potentially universal mechanism for their attachment to host cells. This underscores the significance of HS in viral infection and highlights its potential importance as a target for the development of new antiviral agents.

**Figure 1:**
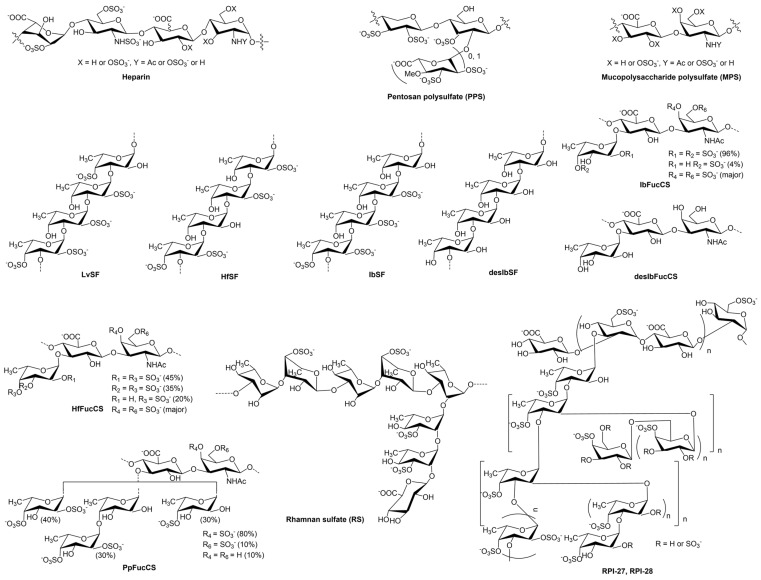
Chemical structures of heparin and the various sulfated glycans studied in this work.

We previously reported a range of sulfated glycans derived from marine sources, which exhibited potent inhibitory activity against SARS-CoV-2 by binding to the viral surface S protein [18,19]. Therefore, this study aims to evaluate the inhibitory activity of sulfated glycans on MERS RBD protein–heparin interactions. We prepared a glycan library, including heparin, heparin-derived oligosaccharides, heparin analogs, and sulfated glycans obtained from marine echinoderms and seaweeds (Figure 1). The inhibitory activity was assessed using Surface Plasmon Resonance (SPR). Our results demonstrated that the MERS RBD protein binds to heparin, and the tested sulfated glycans exhibited strong inhibition of the interaction between heparin and the MERS RBD protein.

## 2. Materials and Methods

### 2.1. Materials

**Figure 2:**
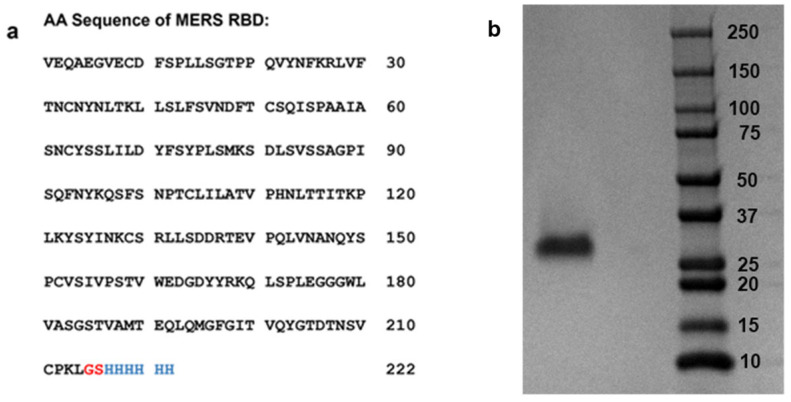
Structure information of MERS-CoV RBD protein. (**a**) Amino acid sequence of MERS-CoV RBD protein (Gly–Ser linker is shown in red, His–tag is shown in blue); (**b**) SDS-PAGE analysis of MERS-CoV RBD (left) shown with protein ladder (right).

Porcine intestinal heparin was purchased from Celsus Laboratories; the average molecular weight (MW) is 15 kDa, and its polydispersity is 1.4 (Cincinnati, OH, USA). A 6-*O*-desulfated heparin (6-Des heparin, MW of 13 kDa) was provided by Dr. Wang from the University of South Florida. The 2-*O*-desulfated IdoA heparin (2-Des heparin, MW of 13 kDa) and *N*-desulfated heparin (*N*-Des heparin, MW of 14 kDa) were prepared according to Yates et al. in our lab. The heparin oligosaccharides, including dp4, dp6, dp8, dp10, dp12, dp14, dp16, and dp18 (dp as degree of polymerization) were purchased from Iduron (Manchester, UK). Pentosan polysulfate (PPS, MW of 6.5 kDa) was from Bene Pharma (Munich, Germany). Mucopolysaccharide polysulfate (MPS, MW of 14.5 kDa) was purchased from Luitpold Pharma (Munich, Germany). Eight marine invertebrate sulfated glycans and derivatives (IbSF, desIbSF, IbFucCS, desIbFucCS, PpFucCS, LvSF, HfSF, HfFucCS) were isolated from the sea cucumbers *Isostichopus badionotus*, *Holothuria floridana*, and *Pentacta pygmaea* and from the sea urchin *Lytechinus variegatus* in Dr. Pomin’s laboratory at the University of Mississippi. RPI-27 and RPI-28, two algal sulfated glycans, were purified in Dr. Jin’s laboratory from the seaweed *Saccharina japonica*. Rhamnan sulfate (RS) was purified from *Monostroma nitidum* by Drs. Linhardt/Zhang group. MERS RBD protein was donated by Dr. Bates’s laboratory at the University of Mississippi. The MERS RBD protein consists of 222 amino acids and has a predicted MW of 30.9 kDa (Figure 2). Sensor streptavidin (SA) chips were purchased from Cytiva (Uppsala, Sweden). SPR measurements were performed on a T200 SPR (Uppsala, Sweden). SPR data processing was conducted using Biaevaluation software (version 3.2).

### 2.2. Expression and Purification of MERS-CoV RBD Protein

An expression construct encoding amino acids 358-571 (VEQA…CPKL) of the MERS S glycoprotein and a carboxy terminal 6x HIS tag was synthesized by Twist Biosciences and cloned into the pTwist CMV BetaGlobin WPRE Neo expression vector. Plasmids were produced in NEB 5-alpha competent *Escherichia coli* (New England Biolabs, Ipswich, MA, USA) and transfected into Expi293 cells using the Expi293 Expression System Kit (Thermo Fisher, Waltham, MA, USA) according to the manufacturer’s instructions. Seven days following transfection, cleared supernatants were passed over a HisTrap HP column (Cytiva), and bound protein was eluted with 450 mM imidazole. Buffer exchange to PBS and protein concentration was performed using centrifugal protein concentrators with a 10 kDa MW cutoff (Pierce, Appleton, WI, USA). Final protein concentration was determined by BCA protein assay (Pierce). MW and protein purity were confirmed by Sodium dodecyl-sulfate polyacrylamide gel electrophoresis (SDS-PAGE) (Bio-Rad, Hercules, CA, USA) (Figure 2).

### 2.3. Preparation of Heparin Biochip

The biotinylated heparin was synthesized according to the following procedure: Dissolve 1 mg of heparin and 1 mg of amine-PEG3-Biotin (Thermo Scientific, Waltham, MA, USA) in 200 μL of water, followed by the addition of 5 mg NaCNBH_3_. The mixture was incubated at 70 °C for 24 h, then another 5 mg NaCNBH_3_ was added, and the reaction continued for an additional 24 h. After completing the reaction, the mixture was desalted using a spin column (3000 molecular weight cut-off). Biotinylated heparin was freeze-dried for chip preparation. For the SPR study, a heparin SA chip was made using the following protocol. Inject a 20 μL solution of biotinylated heparin (0.1 mg/mL) in HBS-EP+ buffer (0.01 M HEPES pH 7.4, 0.15 M NaCl, 3 mM EDTA, 0.05% *v/v* Surfactant P20) (Cytiva, Uppsala, Sweden) over flow cells 2, 3, and 4 of the SA chips at a flow rate of 10 μL/min. Similarly, biotin was immobilized on flow cell 1 as a control channel.

### 2.4. Binding Kinetics and Affinity Studies of the Interaction between Heparin and the MERS RBD Protein

The MERS RBD protein was diluted with HBS-EP+ buffer (pH 7.4). Various dilutions of MERS RBD protein were injected at a flow rate of 30 μL/min. Following each injection, the HBS-EP+ buffer was passed over the sensor surface to perform dissociation for a duration of 180 s. Regeneration of the SPR chip was achieved by injecting 30 μL of 2 M NaCl. The response was continuously monitored as a sensorgram at 25 °C.

### 2.5. Inhibition Activity of the Sulfated Glycans and Marine Sulfated Glycans on Heparin–MERS RBD Protein Interactions

To evaluate the inhibition of the interaction between the MERS RBD protein and heparin, 1000 nM of MERS RBD protein was premixed with different glycans in HBS-EP+ buffer (pH 7.4) and injected over the heparin chip with a flow rate of 30 μL/min. A 30 μL injection of 2 M NaCl was used to regenerate the sensor surface. MERS RBD proteins were used in the control experiments to make sure the surface was completely regenerated. When the binding sites of MERS RBD proteins were occupied by glycan samples, the binding of premixed proteins with glycan samples on the heparin immobilized chip surface was decreased (binding signal (RU) decreased).

### 2.6. Statistical Analysis

Statistical analyses were performed by using one-way ANOVA and Tukey test for multiple comparisons. Graph Pad Prism 9 software was employed for statistical analysis in this study. Statistical significance is defined as *p* > 0.05 (ns), *p* < 0.05 (*/#), *p* < 0.01 (**/##), *p* < 0.001 (***/###), and *p* < 0.0001 (****/####).

## 3. Results and Discussion

### 3.1. Binding Kinetics and Affinity of the Interaction between Heparin and MERS RBD Protein

Like SARS-CoV, MERS-CoV is a zoonotic virus that is transmitted from animals to humans. MERS-CoV shares approximately 57% of its genome homology with SARS-CoV-2 [20]. In alignment with the genomic structure observed in other coronaviruses, the genome of MERS-CoV comprises a single, positive-stranded RNA that encodes a minimum of 10 open reading frames (ORFs). Nine of these ORFs are expressed through seven subgenomic mRNAs and then translated into four primary structural proteins: spike, envelope, membrane, and nucleocapsid. MERS-CoV enters the host cell by binding the viral particle to the host cell surface through the RBD in the S protein interacting with the cellular receptor DPP4 [21]. This underscores the significance of the MERS RBD as a pivotal target in the development of prophylactic MERS-CoV vaccines.

The HSPGs play an important role in facilitating the viral infection of host cells, and the molecular mechanisms of infection can fall within different categories [15]. For example, certain viruses utilize their surface basic residues or capsid proteins interact with the negatively charged sulfated HS chains, and these electrostatic interactions increase the concentration of viruses at the host cell surface, enhancing binding to specific entry receptors. In some other cases, HSPGs may act as primary receptors for the virus. Numerous other viruses leverage HSPG-mediated endocytosis as a mechanism to enter host cells. The endocytic pathways regulated by HSPGs and employed by the virus for cellular entry encompass clathrin-mediated uptake, caveolae/cholesterol-dependent endocytosis, and macropinocytosis. In other cases, for instance, viruses do not initially rely on binding to HSPGs for attachment and the infection of host cells but may acquire HSPGs dependence through intra-host or cell culture adaptation. Hence, pharmaceutical agents directed at HSPGs emerge as promising candidates for disrupting various stages of the viral lifecycle. Notably, heparin, functioning as an HS mimetic, has been shown to competitively inhibit the binding of viral proteins to HS on the host cell surface in numerous instances, particularly against SARS-CoV-2 [22].

**Figure 3:**
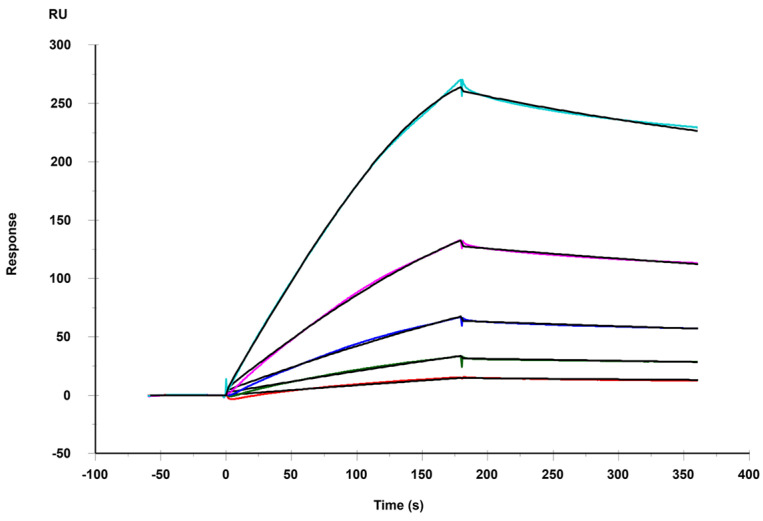
SPR sensorgrams of MERS RBD protein binding with heparin. Concentrations of MERS RBD protein were 1000, 500, 250, 125, 62.5, and 31.3 nM (from top to bottom, respectively). The black curves are the fitting curves using a model from T200 Evaluation software (version 3.2).

Here, a SPR chip coated with heparin was prepared as a surface to assess the binding affinities with MERS RBD. The sensorgrams illustrating the interactions between heparin and the MERS RBD protein are shown in Figure 3. The binding kinetics and affinity, characterized by the association rate constant (k_a_), dissociation rate constant (k_d_), and binding equilibrium dissociation constant (*K_D_*, *K_D_* = k_d_/k_a_), were determined by globally fitting the complete association and dissociation phases using a 1:1 Langmuir binding model. Table 1 presents the kinetic parameters for the interaction between the MERS RBD protein and heparin. The binding kinetic analysis revealed a strong binding affinity with a *K_D_* of 29.4 nM for the MERS RBD–heparin interaction.

### 3.2. SPR Solution Competition between Surface-Immobilized Heparin and Heparin Oligosaccharides and Desulfated Heparins

**Figure 4:**
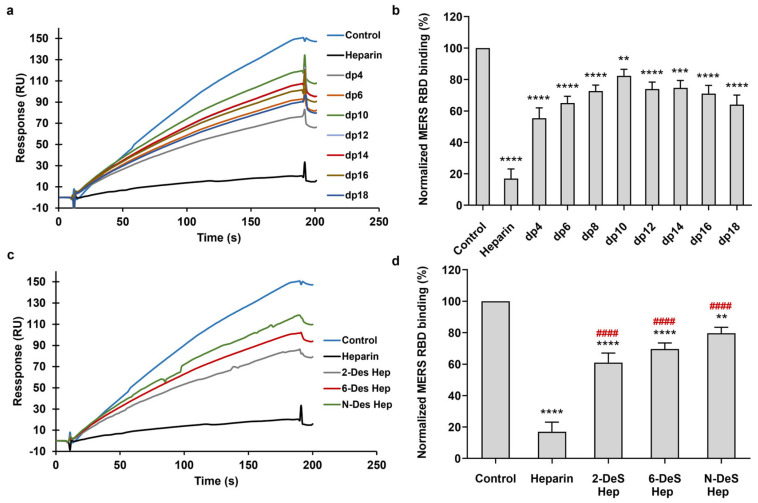
MERS RBD protein–heparin interaction inhibited by heparin oligosaccharides and desulfated heparins using solution competition. (**a**) SPR sensorgrams of MERS RBD protein–heparin interaction competing with different heparin oligosaccharides. (**b**) Bar graphs (based on triplicate experiments with standard deviation) of MERS RBD protein binding preference to surface heparin by competing with different heparin oligosaccharides. (**c**) SPR sensorgrams of MERS RBD protein–heparin interaction competing with different desulfated heparins. (**d**) Bar graphs (based on triplicate experiments with standard deviation) of normalized MERS RBD protein binding preference to surface heparin by competing with different desulfated heparins. Data are shown as means ± SD and are analyzed using a one-way ANOVA/Tukey tests (* in black indicates comparison to the control, # in red indicates comparison to the heparin).

We used oligosaccharide chains with varying degrees of polymerization (from dp4 to dp18) to investigate the influence of oligosaccharide chain length on the interaction between heparin and the MERS RBD protein. We performed solution/surface competition SPR experiments to assess the inhibitory effects of various heparin oligosaccharides on the interaction between heparin (on the surface) and the MERS RBD. The MERS RBD protein solution (1000 nM) was individually pre-mixed with an equivalent concentration (1000 nM) of each heparin oligosaccharide. Solution competition analyses between heparin and heparin oligosaccharides are presented in Figure 4a. All heparin oligosaccharides demonstrated inhibition of MERS RBD binding to the heparin-coated surface, as compared to the control (Figure 4b). Heparin exhibited an 83% inhibition in the binding of the MERS RBD to the surface-immobilized heparin. Oligosaccharides of heparin, ranging from dp4 to dp18, demonstrated inhibitions of binding ranging from 18% to 45%, with no apparent dependence on glycan length.

Furthermore, to elucidate the structural determinants contributing to heparin competition, we evaluated the capacity of various chemically desulfated heparins (with comparable chain lengths) to inhibit the interaction between the MERS RBD protein and surface-immobilized heparin. All three desulfated heparins (2-Des heparin, 6-Des heparin, and *N*-Des heparin) exhibited a reduction in the binding of MERS RBD to surface-immobilized heparin, as compared to the heparin control (Figure 4c,d). Hence, the binding affinity between the MERS RBD protein and heparin was minimized by the removal of any sulfate from heparin. Nevertheless, the distinctions among the three desulfated samples did not reach statistical significance, indicating that binding specificity was not dictated by the sulfate group positions but rather primarily relied on the presence of sufficient charge.

### 3.3. Inhibition of PPS and MPS on the Interaction between Heparin and MERS RBD Protein

PPS [23] is produced by chemically sulfonating a β-(1→4)-xylan derived from plants, featuring a highly sulfated polysaccharide backbone (Figure 1). The MW of PPS ranges from 4.0 to 6.0 kDa, close to the MW of low-MW heparins. It serves as a heparin mimetic and has been used as an antithrombotic agent in clinical settings in the United States and has been extensively studied across various clinical disorders, including the antagonism of enzymatic activities and the inhibition of HIV infectivity.

MPS [24], a GAG with a chemical structure similar to heparin, is referred to as a heparinoid. MPS is a semi-synthetic glycosaminoglycan initial isolation from mammalian cartilage and subsequent chemical sulfation. The structure of MPS is composed of repetitive units comprising monomeric amino sugars and uronic acid, forming an unbranched structure. The average MW of MPS is around 9.7 kDa. MPS has been utilized in the topical treatment of superficial phlebitis, hematomas, and sports-related injuries.

**Figure 5:**
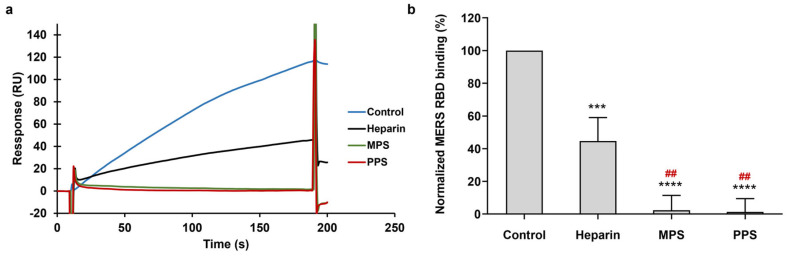
Solution competition between heparin and MPS or PPS. (**a**) SPR sensorgrams of MERS RBD protein–heparin interaction competing with MPS or PPS. (**b**) Bar graphs (based on triplicate experiments with standard deviation) of normalized MERS RBD protein binding preference to surface heparin by competing with PPS or MPS. Data are shown as mean ± SD and are analyzed using a one-way ANOVA/Tukey tests (* in black indicates comparison to the control, # in red indicates comparison to the heparin).

To test the inhibitory potential of two types of heparin analogs on the interaction between heparin and the MERS RBD protein, a mixture of 1000 nM of the MERS RBD protein with the same concentrations of PPS, MPS, and heparin (10 μg/mL) was prepared. Figure 5a illustrates the solution competition between heparin and multiple sulfated glycans. In comparison to the positive control (heparin in solution), both PPS and MPS in the solution demonstrated heightened inhibitory activity against the interaction of surface-immobilized heparin with the MERS RBD. PPS and MPS exhibited forceful inhibitions of the MERS RBD–heparin interaction, with inhibitory rates of 98% and 97% (Figure 5b), respectively. This observation may be attributed to the higher degree of sulfation in MPS and PPS when compared to that of heparin. The average disaccharide unit in heparin comprises approximately 2.7 sulfate groups, whereas the disaccharide unit in PPS contains more than three sulfate groups, and in MPS, it has more than four sulfate groups. The high sulfate group content facilitates the interaction between glycans and the S protein. These results suggest that PPS and MPS show promise as potential candidates for therapeutic or preventive agents against MERS-CoV infection.

### 3.4. Inhibition of IbSF/IbFucCS on the Interaction between Heparin and MERS RBD Protein

The marine sulfated glycans under investigation included IbSF and IbFucCS [25,26], along with their fully desulfated chemical derivatives desIbSF and desIbFucCS. IbSF and IbSFucCS were extracted from the sea cucumber *I. badionotus*. The structure of IbSF consists of the repeating unit [→3)-α-Fuc2,4S-(1→3)-α-Fuc2S-(1→3)-α-Fuc2S-(1→3)-α-Fuc-(1→], and IbFucCS’s structural repeating unit is [→3)-β-GalNAc4,6S-(1→4)-β-GlcA[(3→1)Y]-(1→], where Y = α-Fuc2,4S (96%) or α-Fuc4S (4%) (Figure 1). Both IbSF and IbFucCS had good anticoagulant and antithrombotic activities.

**Figure 6:**
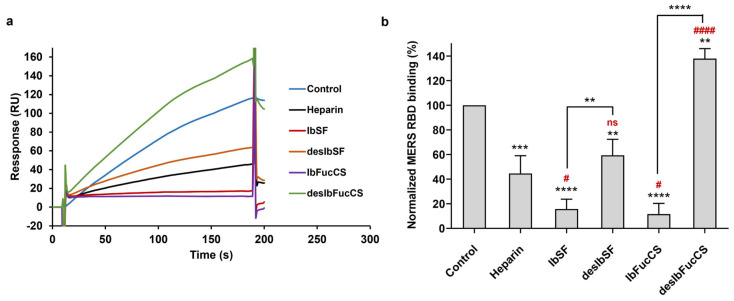
Solution competition between heparin and Ib glycans. (**a**) SPR sensorgrams of MERS RBD protein–heparin interaction competing with IbSF, IbFucCS, desIbSF, and desIbFucCS. (**b**) Bar graphs (based on triplicate experiments with standard deviation) of normalized MERS RBD protein binding preference to surface heparin by competing with different Ib glycans. Data are shown as means ± SD and are analyzed using a one-way ANOVA/Tukey tests (* in black indicates comparison to the control, IbSF with desulfated IbSF, and IbFFuCS with desulfated IbFuCS; # in red indicates comparison to the heparin).

In our previous study, these two marine-derived sulfated glycans demonstrated high potent inhibitory activity against both the wild-type and Delta strains of another coronavirus, SARS-CoV-2 [22]. This inhibition was achieved by effectively interfering with the interaction between the S protein and HS on the host cell surface [27]. Solution/surface competition experiments were used to investigate the inhibitory potential of these two marine sulfated glycans and their fully desulfated derivatives on the interaction between heparin and the MERS RBD protein. The Ib glycans, at an identical concentration of 10 μg/mL, were individually premixed with MERS RBD protein solution (1000 nM) before the injection. All the Ib glycans demonstrated inhibition of the interaction between the MERS RBD protein and surface-immobilized heparin (Figure 6a,b). Notably, both IbSF and IbFucCS displayed highly effective competitive inhibitions compared to that of soluble heparin, achieving rates of 84% and 88%, respectively. Upon complete desulfation, both desIbSF and desIbFucCS exhibited reduced competitive capabilities in inhibiting heparin binding to the MERS RBD. These observations suggest that IbSF and IbSFucCS could possess potential anti-MERS activity, and sulfation is a crucial structural element in marine sulfated glycans for interactions with the MERS RBD protein.

### 3.5. Inhibition of HfSF/HfFucCS; LvSF, PpFucCS, and Seaweed Sourced Glycans (RPI-27, RPI-28, and RS) on the Interaction between Heparin and MERS RBD Protein

HfSF and HfFucCS [28] are sulfated polysaccharides isolated from the sea cucumber *H. floridana* (Hf). HfSF, a sulfated fucan, has the structure [→3)-α-Fuc2,4S-(1→3)-α-Fuc-(1→3)-α-Fuc2S-(1→3)-α-Fuc2S-(1→]n. HfFucCS is a chondroitin sulfate with fucose substitution, featuring the subsequent structure of [→3)-β-GalNAc4,6S-(1→4)-β-GlcA-[(3→1)Y]-(1→]n, where Y = αFuc2,4S (45%), α-Fuc3,4S (35%), or α-Fuc4S (20%) (Figure 1). Sulfated fucan LvSF [29] is isolated from the sea urchin *L. variegatus*, with a repeating sequence of [→3)-α-Fuc2,4S-(1→3)-α-Fuc2S-(1→3)-α-Fuc2S-(1→3)-α-Fuc4S-(1→]n. PpFucCS [19], a fucosylated chondroitin sulfate, is derived from the sea cucumber *P. pygmaea*. It features a structure of [→3)-β-GalNAcX(1→4)-β-GlcA-[(3→1)Y]-(1→]n, where X = 4S (80%), 6S (10%), or non-sulfated (10%); and Y = α-Fuc2,4S (40%), α-Fuc2,4S(1→4)-α-Fuc (30%), or α-Fuc4S (30%) (Figure 1).

**Figure 7:**
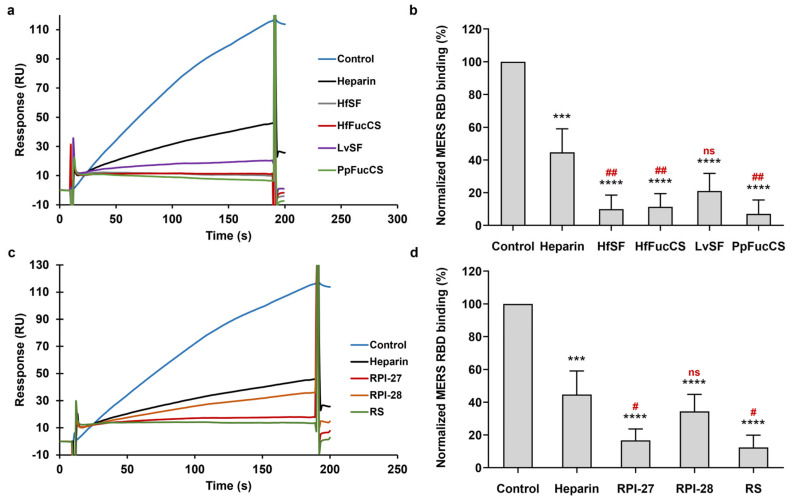
Solution competition between heparin and marine-soured sulfated glycans. (**a**) SPR sensorgrams of MERS RBD protein–heparin interaction competing with sulfated glycans derived from marine invertebrates. (**b**) Bar graphs (based on triplicate experiments with standard deviation) of normalized MERS RBD protein binding preference to surface heparin by competing with different sulfated glycans derived from marine invertebrates. (**c**) SPR sensorgrams of MERS RBD protein–heparin interaction competing with sulfated glycans isolated from seaweeds. (**d**) Bar graphs (based on triplicate experiments with standard deviation) of normalized MERS RBD protein binding preference to surface heparin by competing with different sulfated glycans derived from sulfated glycans isolated from seaweeds. Data are shown as means ± SD and are analyzed using a one-way ANOVA/Tukey tests (* in black indicates comparison to the control, # in red indicates comparison to the heparin).

We used solution/surface competition SPR experiments to investigate the ability of HfSF, HfFucCS, LvSF, and PpFucCS to inhibit the interactions between the MERS RBD and immobilized heparin. The MERS RBD protein solution (1000 nM) was individually mixed with each of these four marine-sourced glycans at the same concentration (10 μg/mL). The results of the solution competition SPR experiments are shown in Figure 7a,b. Heparin demonstrated a 55% inhibition of the binding of the MERS RBD protein to surface-immobilized heparin. HfSF and HfFucCS exhibited remarkable inhibitory activity in preventing the binding of the MERS RBD to surface-immobilized heparin, with rates of 90% and 88%, respectively. PpFucCS and LvSF also showed significant inhibitory effects on the binding of the MERS RBD protein to surface-immobilized heparin, with rates of 93% and 79%, respectively.

The fucoidans labeled as RPI-27 and RPI-28 [30] belong to a class of sulfated heteropolysaccharides obtained from the brown seaweed *S. japonica*. Both RPI-27 and RPI-28 exhibit a highly branched structure and possess two kinds of polysaccharide backbones: (1) a sulfated glucuronomannan and a glucuronomannan backbone featuring repeating 4-linked GlcA and 2-linked mannose, along with a Man residue containing the first C-6 sulfated mannopyranose residue from the nonreducing terminus; (2) a glucuronan with a backbone of 3-linked GlcA. Additional branched chains include GlcA-(1→3)-Man-(1→4)-GlcA, Man-(1→3)-GlcA-(1→4)-GlcA, Fuc-(1→4)-GlcA, and Fuc-(1→3)-Fuc (Figure 1). The average MWs of RPI-27 and RPI-28 were 100 kDa and 12 kDa, respectively. RPI-27 and RPI-28 have demonstrated significant antiviral activity against SARS-CoV-2 in vitro, surpassing the effectiveness of remdesivir (a nucleoside analogue prodrug) [31].

RS [18,32], discovered in a green seaweed *M. nitidum*, is a rhamnose-predominant, high-molecular-weight glycan fraction with high sulfation. The RS backbone is primarily composed of repeating units [→3)-α-L-Rhap-(1→, →2)-α-L-Rhap-(1→, and →2,3)-α-L-Rhap-(1→], with significant sulfation occurring at the C4 hydroxyl groups. RS has various bioactivities, including antioxidative, antihyaluronidase, antitumor, anti-obesity, anti-hypercholesterolemic, anti-hyperglycemic, and anti-diabetic properties [18,33].

In this competition SPR analysis, the same concentration of seaweed-sourced glycans (10 μg/mL) was mixed with 1000 nM of MERS RBD individually. The solution competition results between these glycans and heparin are shown in Figure 7c,d. RPI-27 and RPI-28 showed notable efficacy in inhibiting the binding of the MERS RBD protein to surface-immobilized heparin. Specifically, RPI-27, with a higher molecular weight, demonstrated a stronger inhibition of 83% compared to RPI-28′s 65%. Furthermore, RS also displayed significant inhibitory effects on the binding of the MERS RBD protein to surface-immobilized heparin, achieving an 87% inhibition.

**Figure 8:**
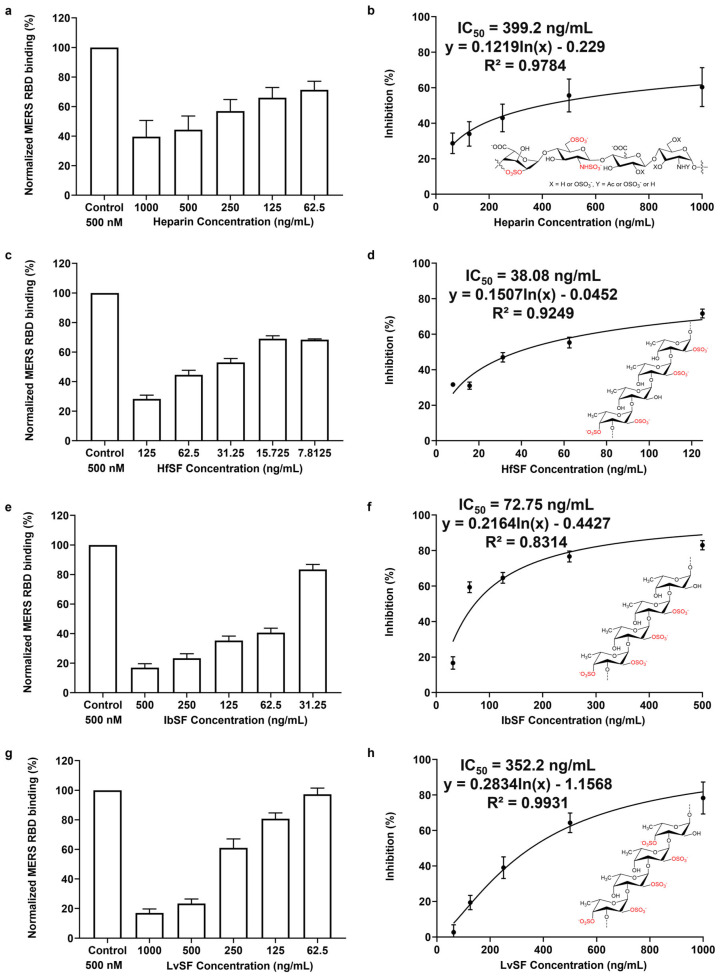
IC50 measurement of the inhibition of MERS RBD binding to heparin using solution competition SPR by sulfated fucans. (**a**,**b**): heparin; (**c**,**d**): HfSF; (**e**,**f**): IbSF; (**g**,**h**): LvSF. IC50 values were calculated using dose–response-inhibition equations in GraphPad Prism 9. Data shown as mean ± SD.

All these nine naturally marine-sourced sulfated glycans (IbSF, IbFucCS, HfSF, HfFucCS, PpFucCS, LvSF, RPI-27, PRI-28, and RS) exhibited the capacity to inhibit the interactions between MERS-CoV S proteins and surface-immobilized heparin. The inhibitory effects of heparin and marine sulfated glycans (LvSF, IbSF, IbFucCS, and PpFucCS) against MERS-CoV have been elucidated in previous studies utilizing a pseudotyped lentiviral particles methodology [34]. The results indicate that these four marine sulfated glycans demonstrate more potent anti-MERS-CoV activity in comparison to that in heparin, aligning with the trends observed in our own investigation. The differential activity observed among various glycans is considered reasonable given that it occurs within two distinct analytical systems. However, the chemically desulfated glycans, both desIbSF and desIbFucCS, displayed significantly reduced binding activity of S proteins to surface-immobilized heparin. Our study demonstrates the critical roles of sulfation in the inhibitory activity of marine sulfated glycans. IbFucCS, HfFucCS, and PpFucCS are three distinct marine-sourced fucosylated chondroitin sulfates with varying degrees of branching disulfated fucoses; the proportions are 96%, 80%, and 70%, respectively. Despite having the lowest sulfation level, PpFucCS exhibited the best inhibitory activity against the MERS RBD protein. FucCS and HfFucCS exhibited similar inhibitory activity levels, both of which were slightly weaker than that of PpFucCS. Fucoidans IbSF, LvSF, and HfSF share a common fucan tetrasaccharide repeating unit but exhibit distinct sulfation patterns. LvSF, with higher sulfation, has pentasulfated tetrasaccharide building blocks, while IbSF and HfSF have tetrasulfated tetrasaccharide building blocks. The inhibitory potencies among these follow the order from strongest to weakest as HfSF, IbSF, then LvSF. The application of polysaccharides from seaweed sources is more cost-effective than that of those from marine invertebrates. Among the polysaccharides sourced from marine invertebrates, sulfated fucans exhibit low anticoagulant effects while maintaining potent antiviral properties compared to those of fucosylated chondroitin sulfate [34]. Therefore, we selected three seaweed-sourced glycans (RPI-27, RPI-28, and RS) and three types of sulfated fucans (HfSF, IbSF, and LvSF) for solution competition dose–response analysis. In the competitive SPR analysis, a series of concentrations of six marine-sourced glycans were individually mixed with 1000 nM MERS RBD. Half-maximal inhibitory (IC_50_) concentrations (50% reduction in RUs) are shown in Table 2 and Figure 8 and Figure S1. Heparin and LvSF demonstrated a comparable modest inhibitory potential, with estimated IC_50_ values around 350 ng/mL. HfSF and IbSF exhibited stronger inhibitory effects, yielding IC_50_ values of 38.1 ng/mL and 72.8 ng/mL, respectively. These results indicate that the sulfation pattern exerts a more significant influence on interactions with MERS RBD than the degree of sulfation.

## 4. Conclusions

The present study demonstrated the strong binding of the MERS RBD protein to heparin. We investigated the binding affinity of sulfated glycans with various structures to the MERS RBD. Solution competition analysis between surface-immobilized heparin and oligo-heparins ranging from dp4 to dp18 revealed that the binding was not length-dependent. Desulfated heparin at different positions showed a lower binding affinity compared to that of heparin, indicating that binding is associated with the density of negative charges. In a competition SPR assay, PPS and MPS in solution exhibited remarkable inhibitory activity against surface-immobilized heparin binding to the MERS RBD. These findings suggest that GAG binding to the MERS RBD is dependent on charge interactions. Solution competition analysis between surface-immobilized heparin and 11 defined marine sulfated glycans (IbSF, desIbSF, IbFucCS, desIbFucCS, HfSF, HfFucCS, PpFucCS, LvSF, RPI 27, PRI 28, and RS) demonstrated that the natural marine sulfated glycans provided outstanding inhibitory activity against surface-immobilized heparin binding to the MERS RBD protein. However, fully desulfated agents desIbSF and desIbFucCS exhibited non-inhibitory activity. The binding appeared to depend on the degree of sulfation and the sulfation pattern. Heparin analogs and the library of natural sulfated glycans sourced from marine organisms have shown its potential for use as a therapeutic agent and/or preventative antiviral drug. Despite the strong inhibitory activity of all the tested sulfated glycans against both viral proteins in the SPR-based binding to surface-immobilized heparin, there are no clear correlations between the structural features of these glycans and their binding properties. Future studies are needed to investigate the structure–activity relationships, bioavailability, and antiviral activity of these sulfated glycans.

## Figures and Tables

**Table 1:** Kinetic data of MERS RBD protein binding with heparin surface.

	k_a_ (M^−1^ S^−1^)	k_d_ (S^−1^)	*K_D_* (M)
MERS RBD	4.43 × 10^4^ (±460) *	1.45 × 10^−3^ (±5.8 × 10^−6^) *	2.94 × 10^−8^ (±4.5 × 10^−9^) **

* The data with (±) in parentheses represent the standard deviation (SD) obtained from the global fitting of five injections. ** SD based on triplicate measurements.

**Table 2:** Summary of IC_50_ measurement between heparin and marine-derived glycans binding to MERS RBD protein.

	Heparin	RPI-27	RPI-28	RS	HfSF	IbSF	Lvsf
IC_50_ (ng/mL)	399.2	21.1	269.4	17.2	38.1	72.8	352.2

## Data Availability

Data are contained within the article and Supplementary Materials.

## Supplementary Materials

The following supporting information can be downloaded at: https://www.mdpi.com/article/10.3390/v16020237/s1, Figure S1: IC_50_ measurement of the inhibition of MERS RBD binding to heparin using solution competition SPR by seaweed-sourced sulfated glycans.

## References

[1] Zaki A.M., Van Boheemen S., Bestebroer T.M., Osterhaus A.D.M.E., Fouchier R.A.M. (2012). Isolation of a Novel Coronavirus from a Man with Pneumonia in Saudi Arabia. N. Engl. J. Med..

[2] De Groot R.J., Baker S.C., Baric R.S., Brown C.S., Drosten C., Enjuanes L., Fouchier R.A.M., Galiano M., Gorbalenya A.E., Memish Z.A. (2013). Commentary: Middle East Respiratory Syndrome Coronavirus (MERS-CoV): Announcement of the Coronavirus Study Group. J. Virol..

[3] MERS Situation Update. https://www.emro.who.int/health-topics/mers-cov/mers-outbreaks.html.

[4] Yang Y., Du L., Liu C., Wang L., Ma C., Tang J., Baric R.S., Jiang S., Li F. (2014). Receptor Usage and Cell Entry of Bat Coronavirus HKU4 Provide Insight into Bat-to-Human Transmission of MERS Coronavirus. Proc. Natl. Acad. Sci. USA.

[5] Gao J., Lu G., Qi J., Li Y., Wu Y., Deng Y., Geng H., Li H., Wang Q., Xiao H. (2013). Structure of the Fusion Core and Inhibition of Fusion by a Heptad Repeat Peptide Derived from the S Protein of Middle East Respiratory Syndrome Coronavirus. J. Virol..

[6] Forni D., Filippi G., Cagliani R., De Gioia L., Pozzoli U., Al-Daghri N., Clerici M., Sironi M. (2015). The Heptad Repeat Region Is a Major Selection Target in MERS-CoV and Related Coronaviruses. Sci. Rep..

[7] Raj V.S., Mou H., Smits S.L., Dekkers D.H.W., Müller M.A., Dijkman R., Muth D., Demmers J.A.A., Zaki A., Fouchier R.A.M. (2013). Dipeptidyl Peptidase 4 Is a Functional Receptor for the Emerging Human Coronavirus-EMC. Nature.

[8] Millet J.K., Whittaker G.R. (2014). Host Cell Entry of Middle East Respiratory Syndrome Coronavirus after Two-Step, Furin-Mediated Activation of the Spike Protein. Proc. Natl. Acad. Sci. USA.

[9] Xia S., Liu Q., Wang Q., Sun Z., Su S., Du L., Ying T., Lu L., Jiang S. (2014). Middle East Respiratory Syndrome Coronavirus (MERS-CoV) Entry Inhibitors Targeting Spike Protein. Virus Res..

[10] Lu L., Liu Q., Zhu Y., Chan K.-H., Qin L., Li Y., Wang Q., Chan J.F.-W., Du L., Yu F. (2014). Structure-Based Discovery of Middle East Respiratory Syndrome Coronavirus Fusion Inhibitor. Nat. Commun..

[11] Xu Y., Lou Z., Liu Y., Pang H., Tien P., Gao G.F., Rao Z. (2004). Crystal Structure of Severe Acute Respiratory Syndrome Coronavirus Spike Protein Fusion Core. J. Biol. Chem..

[12] Liang R., Wang L., Zhang N., Deng X., Su M., Su Y., Hu L., He C., Ying T., Jiang S. (2018). Development of Small-Molecule MERS-CoV Inhibitors. Viruses.

[13] Du L., Zhao G., Kou Z., Ma C., Sun S., Poon V.K.M., Lu L., Wang L., Debnath A.K., Zheng B.-J. (2013). Identification of a Receptor-Binding Domain in the S Protein of the Novel Human Coronavirus Middle East Respiratory Syndrome Coronavirus as an Essential Target for Vaccine Development. J. Virol..

[14] Parag S., Carnevale K. (2022). COVID-19 Pathogen Viral Evolution Leading to Increased Infectivity. Cureus.

[15] De Pasquale V., Quiccione M.S., Tafuri S., Avallone L., Pavone L.M. (2021). Heparan Sulfate Proteoglycans in Viral Infection and Treatment: A Special Focus on SARS-CoV-2. Int. J. Mol. Sci..

[16] Ling J., Li J., Khan A., Lundkvist Å., Li J.-P. (2022). Is Heparan Sulfate a Target for Inhibition of RNA Virus Infection?. Am. J. Physiol.-Cell Physiol..

[17] Hao W., Ma B., Li Z., Wang X., Gao X., Li Y., Qin B., Shang S., Cui S., Tan Z. (2021). Binding of the SARS-CoV-2 Spike Protein to Glycans. Sci. Bull..

[18] Song Y., He P., Rodrigues A.L., Datta P., Tandon R., Bates J.T., Bierdeman M.A., Chen C., Dordick J., Zhang F. (2021). Anti-SARS-CoV-2 Activity of Rhamnan Sulfate from *Monostroma nitidum*. Mar. Drugs.

[19] He P., Shi D., Li Y., Xia K., Kim S.B., Dwivedi R., Farrag M., Pomin V.H., Linhardt R.J., Dordick J.S. (2023). SPR Sensor-Based Analysis of the Inhibition of Marine Sulfated Glycans on Interactions between Monkeypox Virus Proteins and Glycosaminoglycans. Mar. Drugs.

[20] Krishnamoorthy S., Swain B., Verma R.S., Gunthe S.S. (2020). SARS-CoV, MERS-CoV, and 2019-nCoV Viruses: An Overview of Origin, Evolution, and Genetic Variations. VirusDisease.

[21] Oh J., Park U., Kim J., Jeon K., Kim C., Cho N.-H., Choi Y.S. (2023). Enhancing Immune Protection against MERS-CoV: The Synergistic Effect of Proteolytic Cleavage Sites and the Fusion Peptide and RBD Domain Targeting VLP Immunization. Front. Immunol..

[22] Shi D., Bu C., He P., Song Y., Dordick J.S., Linhardt R.J., Chi L., Zhang F. (2022). Structural Characteristics of Heparin Binding to SARS-CoV-2 Spike Protein RBD of Omicron Sub-Lineages BA.2.12.1, BA.4 and BA.5. Viruses.

[23] Bertini S., Alekseeva A., Elli S., Pagani I., Zanzoni S., Eisele G., Krishnan R., Maag K.P., Reiter C., Lenhart D. (2022). Pentosan Polysulfate Inhibits Attachment and Infection by SARS-CoV-2 In Vitro: Insights into Structural Requirements for Binding. Thromb. Haemost..

[24] Hoppensteadt D.A., Neville B., Schultz C., Jeske W., Raake W., Fareed J. (2010). Comparative Studies on the Topical Administration of Mucopolysaccharide and Heparin Ointments in Nonhuman Primates. Clin. Appl. Thromb. Hemost..

[25] Chen S., Xue C., Yin L., Tang Q., Yu G., Chai W. (2011). Comparison of Structures and Anticoagulant Activities of Fucosylated Chondroitin Sulfates from Different Sea Cucumbers. Carbohydr. Polym..

[26] Chen S., Hu Y., Ye X., Li G., Yu G., Xue C., Chai W. (2012). Sequence Determination and Anticoagulant and Antithrombotic Activities of a Novel Sulfated Fucan Isolated from the Sea Cucumber Isostichopus Badionotus. Biochim. Biophys. Acta (BBA) Gen. Subj..

[27] Abdulsalam H., Li J., Loka R.S., Sletten E.T., Nguyen H.M. (2023). Heparan Sulfate-Mimicking Glycopolymers Bind SARS-CoV-2 Spike Protein in a Length- and Sulfation Pattern-Dependent Manner. ACS Med. Chem. Lett..

[28] Shi D., Qi J., Zhang H., Yang H., Yang Y., Zhao X. (2019). Comparison of Hydrothermal Depolymerization and Oligosaccharide Profile of Fucoidan and Fucosylated Chondroitin Sulfate from Holothuria Floridana. Int. J. Biol. Macromol..

[29] Pomin V.H., Pereira M.S., Valente A.-P., Tollefsen D.M., Pavão M.S.G., Mourão P.A.S. (2005). Selective Cleavage and Anticoagulant Activity of a Sulfated Fucan: Stereospecific Removal of a 2-Sulfate Ester from the Polysaccharide by Mild Acid Hydrolysis, Preparation of Oligosaccharides, and Heparin Cofactor II–Dependent Anticoagulant Activity. Glycobiology.

[30] Jin W., Wang J., Ren S., Song N., Zhang Q. (2012). Structural Analysis of a Heteropolysaccharide from *Saccharina japonica* by Electrospray Mass Spectrometry in Tandem with Collision-Induced Dissociation Tandem Mass Spectrometry (ESI-CID-MS/MS). Mar. Drugs.

[31] Wang Y., Zhang D., Du G., Du R., Zhao J., Jin Y., Fu S., Gao L., Cheng Z., Lu Q. (2020). Remdesivir in Adults with Severe COVID-19: A Randomised, Double-Blind, Placebo-Controlled, Multicentre Trial. Lancet.

[32] Yamashiro Y. (2017). Anticoagulant Activity of Rhamnan Sulfate Isolated from Commercially Cultured *Monostroma nitidum*. Int. J. Biomed. Mater. Res..

[33] Suzuki K., Terasawa M. (2020). Biological Activities of Rhamnan Sulfate Extract from the Green Algae *Monostroma nitidum* (Hitoegusa). Mar. Drugs.

[34] Zoepfl M., Dwivedi R., Kim S.B., McVoy M.A., Pomin V.H. (2023). Antiviral Activity of Marine Sulfated Glycans against Pathogenic Human Coronaviruses. Sci. Rep..

