# Supplementary material for: Sulfated Glycans Inhibit the Interaction of MERS-CoV Receptor Binding Domain with Heparin
Source: Viruses. 2024 Feb 2;16(2):237. doi: 10.3390/v16020237 (PMC10892611; doi:10.3390/v16020237)
Supplement: Supplementary file 1 [file viruses-16-00237-s001.zip › viruses-2823707-supplementary.pdf]

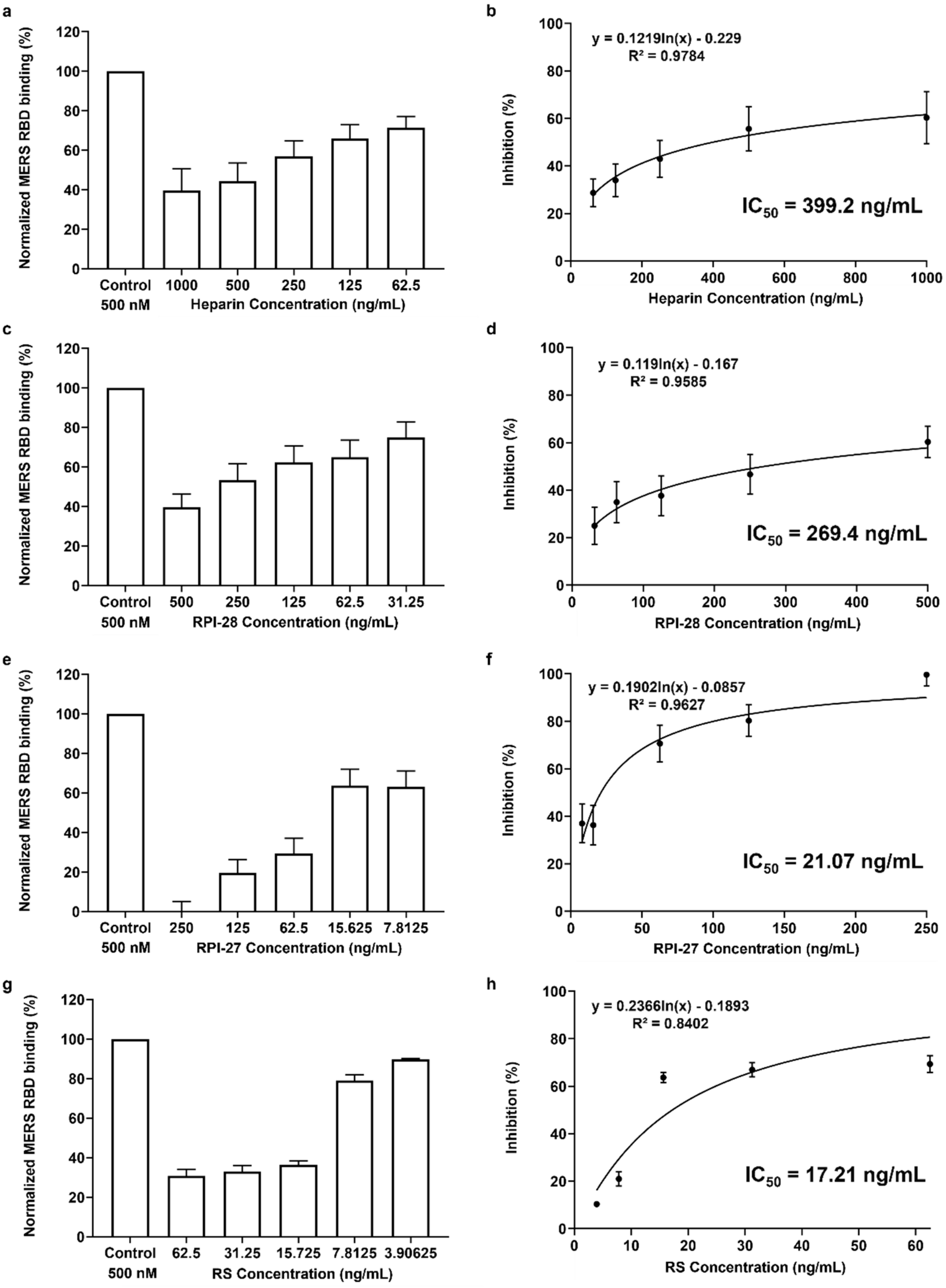

Figure S1. IC_{50} measurement of the inhibition of MERS RBD binding to heparin using solution competition SPR by by seaweed-sourced sulfated glycans. (a, b) = heparin; (c, d) = RPI-28; (e, f) = RPI-27; (g, h) = RS. IC_{50} values were calculated using dose-response-inhibition equations in GraphPad Prism 9. Data showed as mean ± SD.
